# Retinitis pigmentosa with or without skeletal abnormalities due to homozygous mutations in the *CWC27* gene: A case report

**DOI:** 10.1097/MD.0000000000036357

**Published:** 2023-12-22

**Authors:** Yang-Fan Qi, Xiaoping Ma, Shuang-Zhu Lin, Wan-Qi Wang, Jia-Yi Li, Qian-Dui Chen, Li Liu

**Affiliations:** a Changchun University of Chinese Medicine, Changchun, Jilin Province, China; b Department of Children Rehabilitation, The First People’s Hospital of Yinchuan, Yinchaun, Ningxia Province, China; c Diagnosis and Treatment Center for Children, Affiliated Hospital of Changchun University of Chinese Medicine, Changchun, Jilin Province, China.

**Keywords:** *CWC27* gene, global developmental delay, retinitis pigmentosa with or without skeletal abnormalities

## Abstract

**Rationale:**

Retinitis pigmentosa with or without skeletal abnormalities (RPSKA) is an autosomal recessive disorder caused by mutations in the *CWC27* gene. Skeletal dysplasia and non-syndromic retinitis pigmentosa are typical manifestations, and most patients present with retinopathy such as retinitis pigmentosa and limited visual field. Its clinical manifestations are complex and diverse, often involving multiple systems. Examples include short finger deformities, peculiar facial features, short stature, and neurodevelopmental abnormalities, and it is easy to misdiagnose clinically, and early diagnosis is crucial for prognosis.

**Patient concerns:**

A 2-year and 2-month-old female child was admitted to the hospital due to “unsteady walking alone and slow reaction for more than half a year.” After admission, the child was found to have delayed motor development, accompanied by special face, abnormal physical examination of the nervous system, cranial MRI Dandy-Walker malformation, considering developmental delay.

**Diagnoses:**

Whole exome sequencing of the family line revealed the presence of a c.617(exon7)C>A pure mutation in the *CWC27* gene in the affected child (this locus has been reported in the clinical literature); the final diagnosis is RPSKA.

**Interventions:**

Unfortunately, there is no specific drug for the disease; we give children rehabilitation training treatment.

**Outcomes:**

During follow-up process we found that children’s condition is better than before.

**Lessons subsections as per style:**

We reported a case of RPSKA caused by mutations in the *CWC27* gene. This study adds to our understanding of the clinical phenotype of *TBL1XR1* mutations and provides a realistic and reliable basis for clinicians.

## 1. Introduction

The *CWC27* gene (OMIM: 617170), located on chromosome 5q12.3, is a splice-like cyclophilic peptidyl-prolyl cis-trans isomerase^[1]^; Phillips et al^[2]^ first reported children with the gene mutation in 1981. Inheritance is autosomal recessive, and common variants include homozygous and complex heterozygous variants. Retinitis pigmentosa with or without skeletal anomalies (RPSKA) (OMIM: 250410) is an autosomal recessive genetic disease caused by mutations in the *CWC27* gene. The disease can involve multiple systems, with skeletal dysplasia and retinopathy as the main manifestations, accompanied by developmental abnormalities. The characteristic retinopathy is manifested as retinitis pigmentosa, restricted visual field, ptosis of eyelid fissure, and thinning of retinal blood vessels.^[3,4]^ We report a case of a homozygous variant of the *CWC27* gene in which onset of stunting was the main manifestation. Our report enriches the understanding of the clinical phenotype of CWC27 mutations and provides a realistic and reliable basis for clinicians.

## 2. Case presentation

A 2-year and 2-month-old female child suffered from gross motor developmental delay after birth. She could stand upright at 2 months, turn over at 4 months, sit at 6 months, and unconsciously utter “baba, mama” at 1 year and 1 month. Sound, crawling at 1 year and 4 months, walking alone at 1 year and 8 months, still walking unsteadily, slow response, can only consciously pronounce “Dad, Mom.” During the period, the child was 8 months old because she could not climb and was treated in our hospital, and the physical examination found that the muscle tone was high, and there was no significant improvement after giving rehabilitation training guidance, and half a month ago she was treated in our hospital because of “unstable walking alone and slow response,” and was admitted to the hospital after the outpatient clinic with the main complaint of “unstable walking alone and slow response for more than half a year.” Current symptoms: the child is unstable alone, slow response, can only consciously make “dad, mother” sound, no cough and fever, no vomiting and diarrhea, diet and sleep, normal stool.

After admission, we asked the children’s birth and growth history in detail; the children were G4P2, 36 + 6 weeks cesarean section, BW 2000 g (−3SD), born with little amniotic fluid, asphyxiation and rescue history, post-natal diagnosis of “respiratory distress, preterm delivery, low birth weight infants, neonatal pneumonia, neonatal hypoglycemia, ventricular, atrial, patent ductus arteriosus, hyperbilirubinemia, renal horseshoe kidney, hearing abnormalities of both ears,” after treatment were better discharged (unknown). The mother had been suffering from “upper respiratory tract infection” for more than 10 days during pregnancy, without special treatment, before birth due to “little amniotic fluid” oxygen for 2 days, and still denied the special situation. After birth, the children’s great movement development lags behind their peers, they can erect at the age of 2 months, turn over at the age of 4 months, sit at the age of 6 months, unconsciously give the sounds of “baba, mama” at the age of 1 year, climb at the age of 1 year, 4 months, walk alone at the age of 1 year, and still walk unstably, have slow reaction, have slow eye contact with people, can express sign language, but can only give the sounds of “Mom and Dad,” are happy to talk to themselves, are happy to open the door, can execute simple instructions, cannot show the inconvenience.

The child’s past physical fitness is weak, denying the history of contact with infectious diseases such as “hepatitis, tuberculosis, typhoid fever,” denying the history of food and drug allergies, denying the history of surgical trauma, and the history of vaccination is normal. The father and mother have been healthy in the past, and the child’s brother has been healthy in the past, denying the family genetic history and denying the family history of developmental delay.

Through detailed physical examination, we found many problems in the child. Weight: 15 kg (+2SD), length: 80 cm (−2SD), head circumference: 50 cm. Slow reaction, slow eye contact, can express sign language, but can only pronounce “Dad, Mom,” likes to talk to himself, likes to open and close doors, can execute simple commands, breathes steadily, has a large head circumference, and a high palate arch, low nose bridge, upturned nostrils, low ear position and back, palm lines through both hands, happy tongue sticking out, no edema of eyelids, no conjunctival hyperemia, bilateral pupils are equal in size and round, sensitive to light reflex. No jaundice, rash, bleeding spots, coffee spots, and depigmentation spots were found on the skin and mucous membranes. The thorax was symmetrical without deformity, and the cardiopulmonary examination showed no abnormalities. Examination of the limbs showed that the distance between the toes was asymmetrical. The thumb and third toe of the right foot overlapped the second and fourth toes, and the second toe of the left foot overlapped the third toe. Muscle strength of limbs was grade V, muscle tension was high, bilateral knee tendon reflexes could be elicited, ankle clonus (+), mask test (+), trunk straight test (+), parachute reflex (−), and no abnormalities were found. Motor development examination: the supine position can be turned to the prone position, and the flexibility is acceptable; the prone position can crawl with all 4 limbs, and the coordination is good; the sitting position can sit upright and twist, and the balance of all parties has been established; standing position: can walk alone, unstable, flattened valgus and external rotation of the knee joint and both feet; hand grasping: the hands occasionally clenched fists, grasped a lot, and occasionally pinched radially, and the rest of the physical examination showed no abnormalities.

**Figure 1.**
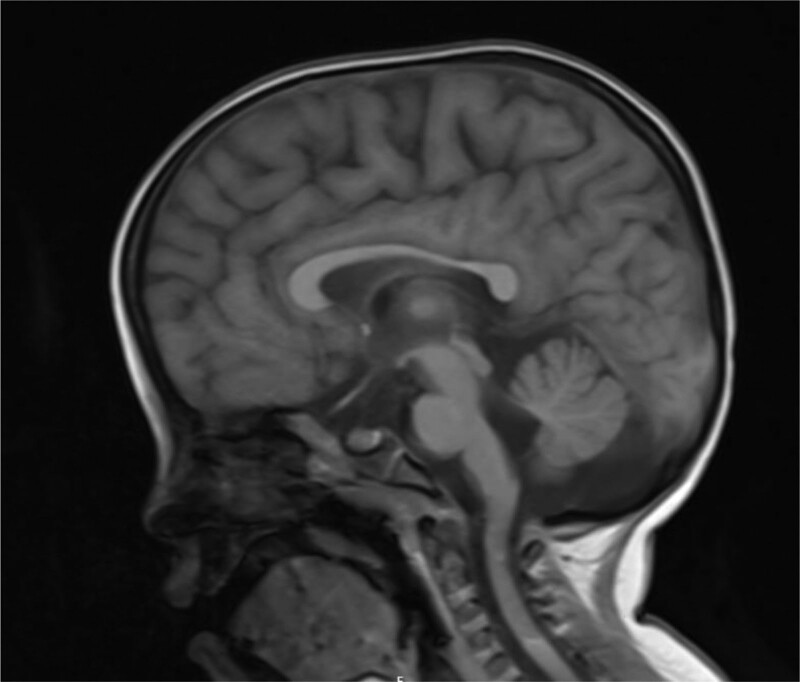
There is a little long T1 and long T2 signal near the left ventricle, and T2 flair shows high signal. Dysplasia of the inferior vermis of the cerebellum, cystic dilatation of the fourth ventricle, and dilatation of the third ventricle and lateral ventricle; no obvious abnormalities were found in the brainstem.

**Figure 2.**
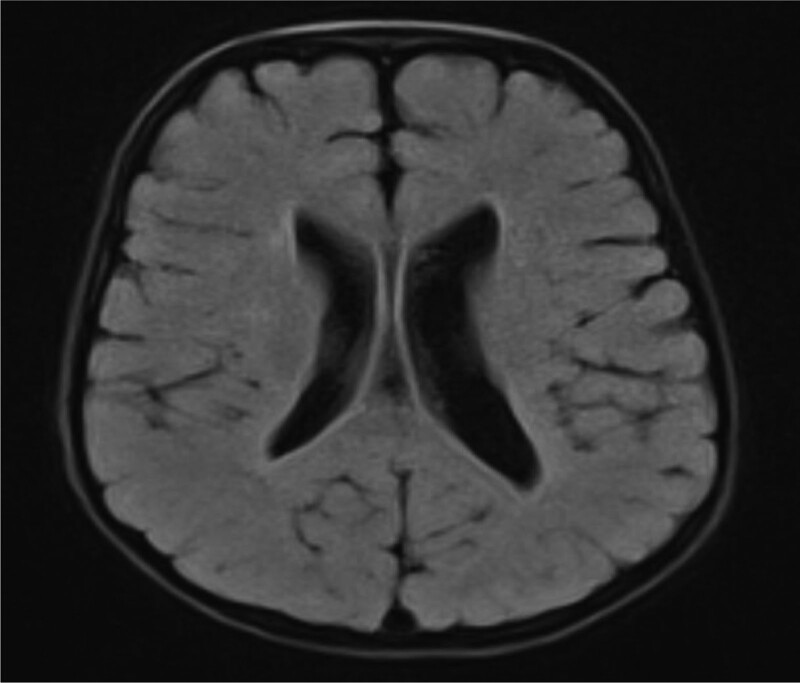
The bilateral frontotemporal extracerebral space is slightly wider, the sulci is deepened, the gyrus is wider, the white matter is reduced, and the midline is centered.

**Figure 3.**
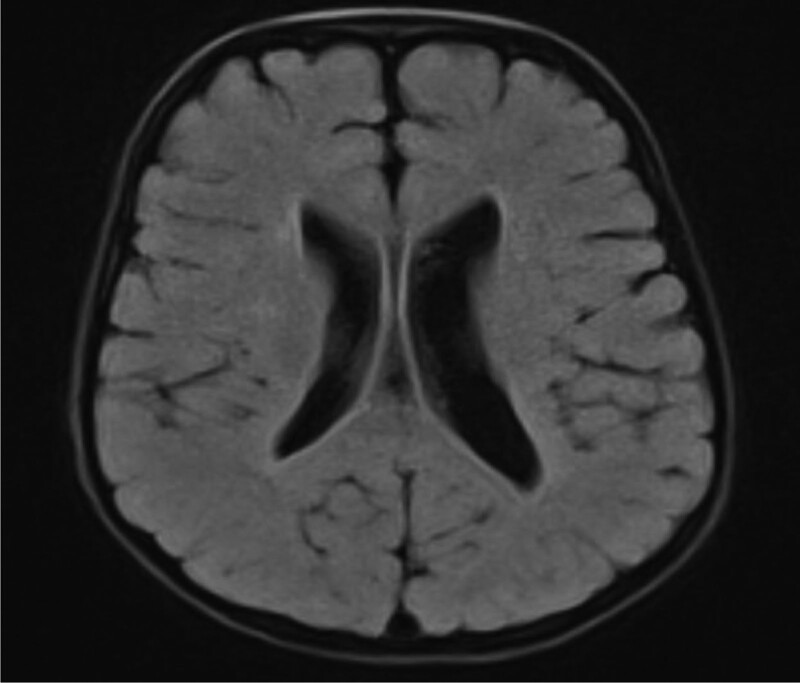
The bilateral frontotemporal extracerebral space is slightly wider, the sulci is deepened, the gyrus is wider, the white matter is reduced, and the midline is centered.

**Figure 4.**
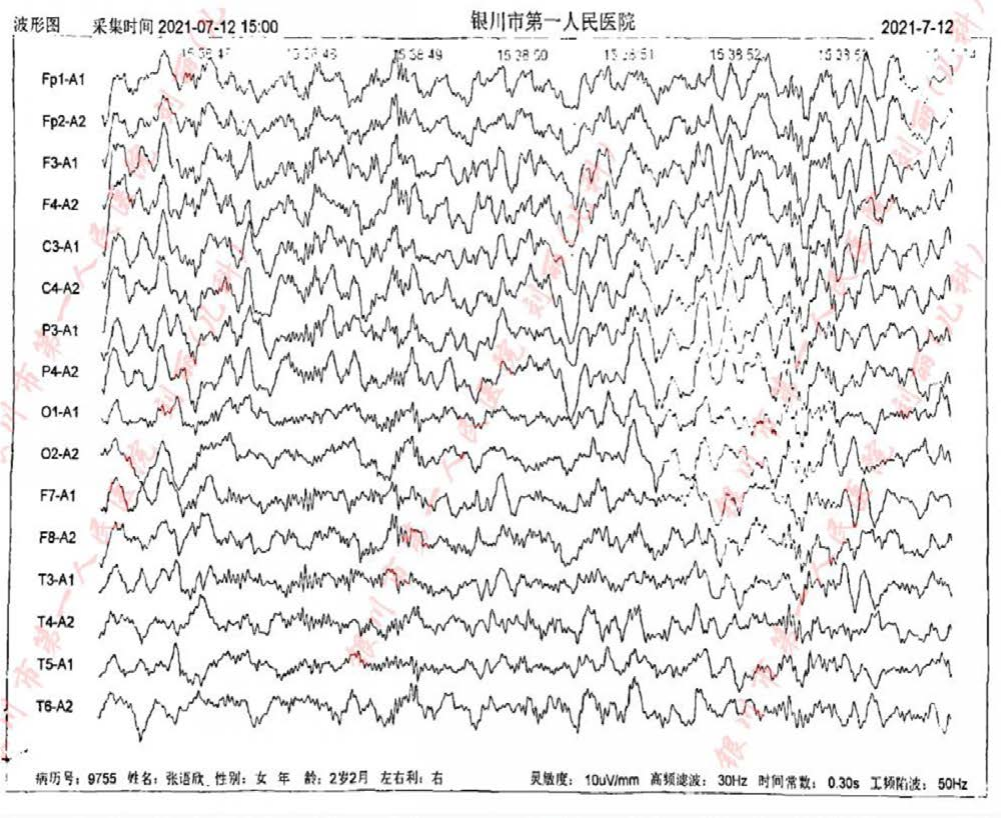
Sleep EEG: medium-high amplitude irregular slow waves can be seen in both leads, mixed with a large number of low-amplitude 18 to 25 Hz β waves, and 12 to 13 Hz sleep spindle waves can be seen, and the left and right are basically symmetrical. Abnormal waves (interictal): a large number of generalized, intermittent, high-amplitude δ short-range paroxyses can be seen during sleep, mainly in the bilateral prefrontal area. During sleep, a large number of medium-to-high amplitude fast wave short bursts can be seen in the bilateral prefrontal area and central area. EEG = electroencephalogram.

**Figure 5.**
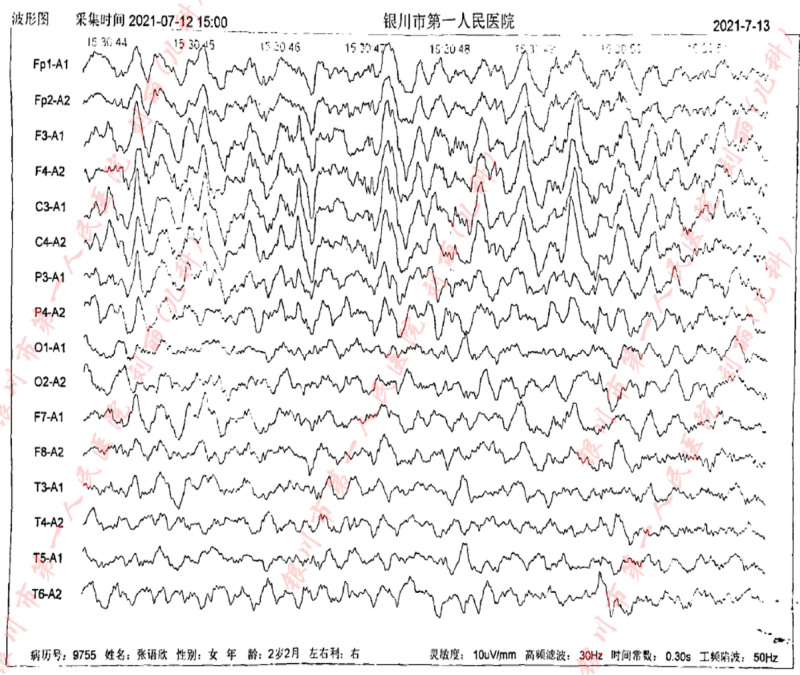
Sleep EEG: medium-high amplitude irregular slow waves can be seen in both leads, mixed with a large number of low-amplitude 18 to 25 Hz β waves, and 12 to 13 Hz sleep spindle waves can be seen, and the left and right are basically symmetrical. Abnormal waves (interictal): a large number of generalized, intermittent, high-amplitude δ short-range paroxyses can be seen during sleep, mainly in the bilateral prefrontal area. During sleep, a large number of medium-to-high amplitude fast wave short bursts can be seen in the bilateral prefrontal area and central area. EEG = electroencephalogram.

**Figure 6.**
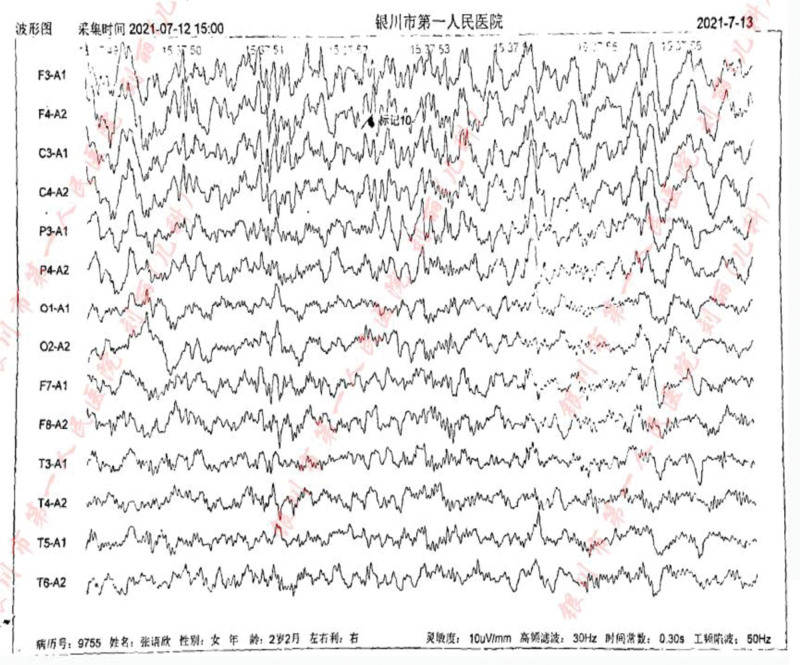
Sleep EEG: medium-high amplitude irregular slow waves can be seen in both leads, mixed with a large number of low-amplitude 18 to 25 Hz β waves, and 12 to 13 Hz sleep spindle waves can be seen, and the left and right are basically symmetrical. Abnormal waves (interictal): a large number of generalized, intermittent, high-amplitude δ short-range paroxyses can be seen during sleep, mainly in the bilateral prefrontal area. During sleep, a large number of medium-to-high amplitude fast wave short bursts can be seen in the bilateral prefrontal area and central area. EEG = electroencephalogram.

**Figure 7.**
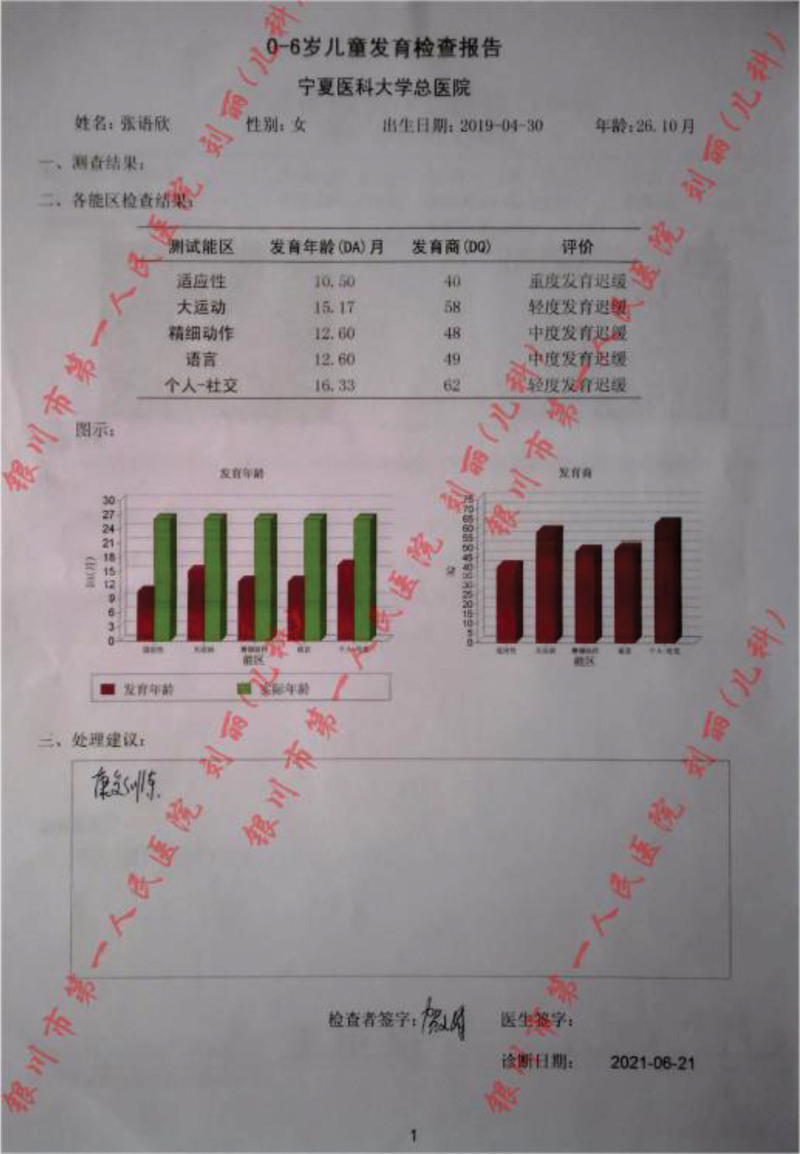
Child developmental screening report for 0–6 year olds.

In addition, we carried out a detailed laboratory test; blood routine, liver function, renal function, and electrolyte test showed no obvious abnormality; thyroid function test: thyroid-stimulating hormone 9.60 IU/mL, the rest showed no obvious abnormality. Echocardiography: aortic coarctation, left ventricular systolic function normal, abdominal ultrasonography: hepatobiliary, pancreatic, splenic, and renal abnormalities. Urologic B-scan showed no obvious abnormality in both kidneys and bladder. Pelvic orthostatic, double upper limbs + double lower limbs full-length X-ray showed no obvious abnormalities in both upper limbs, lower limbs and pelvis, please combine with clinical follow-up and reexamination. Brain magnetic resonance plain scan: 1. Dandy-Walker malformation. 2. The extracerebral space in bilateral frontotemporal regions is slightly wider, the brain sulci are deepened, the ventricles are enlarged, the space between the brains is wider, and the white matter of the brain is reduced. Considering developmental delay, please review MRI in combination with clinical follow-up (Figs. 1–3). Electroencephalogram: pediatric abnormal monitoring electroencephalogram: 1, δ wave paroxysm; 2, bilateral forehead and central area fast wave short array issuance; 3, non-drug fast wave increase (Figs. 4–6). Growth examination report for children aged 0–6 years: adaptive 40 severe developmental delay; major exercises 58 mild developmental delay; fine motor 48 moderate developmental delay; language 49 moderate developmental delay; individual-social 62 mild developmental delay (Fig. 7).

Female children, chronic onset, with developmental retardation as the main manifestation, accompanied by special appearance, abnormal muscle tone, abnormal neurological examination, cranial MRI scan 1, Dandy-Walker malformation. 2. Bilateral frontotemporal extracerebral space is slightly wider, cerebral sulcus deepens, ventricle enlargement, brain width is wider, white matter is reduced, considering developmental retardation, and the onset of children is young, we highly suspect genetic diseases caused by gene mutations.

**Figure 8.**
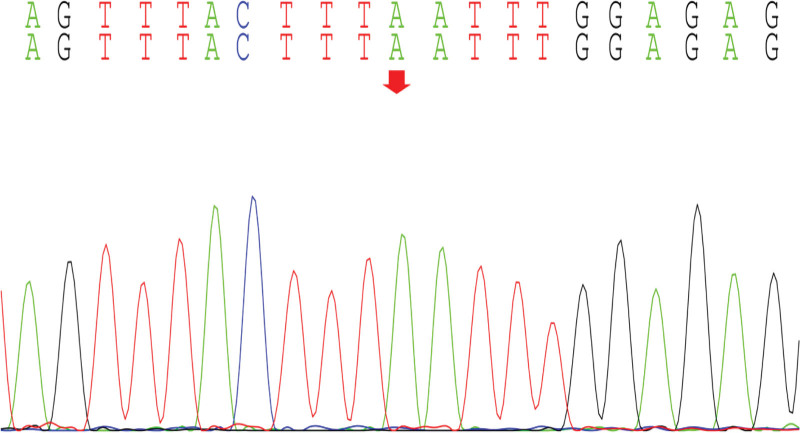
CWC27: c.617 (exon7) C>A (p.Ser206Ter): Proband.

**Figure 9.**
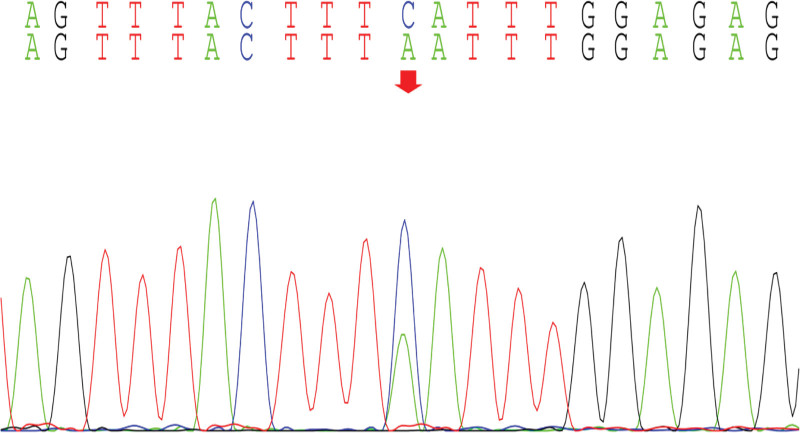
Father of the proband.

**Figure 10.**
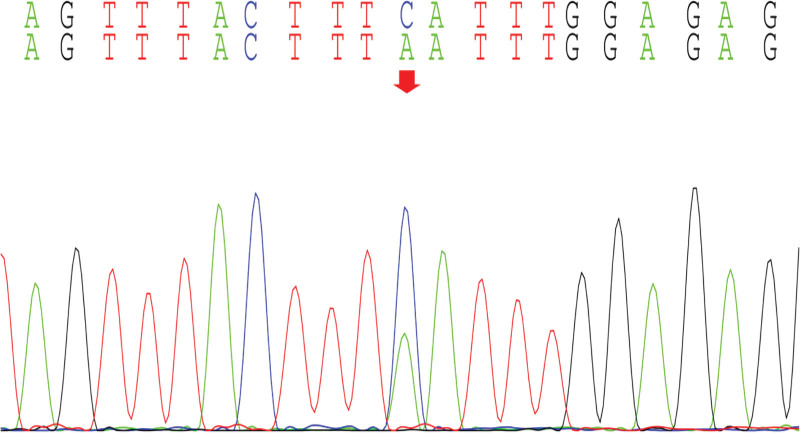
Mother of the proband.

**Figure 11.**
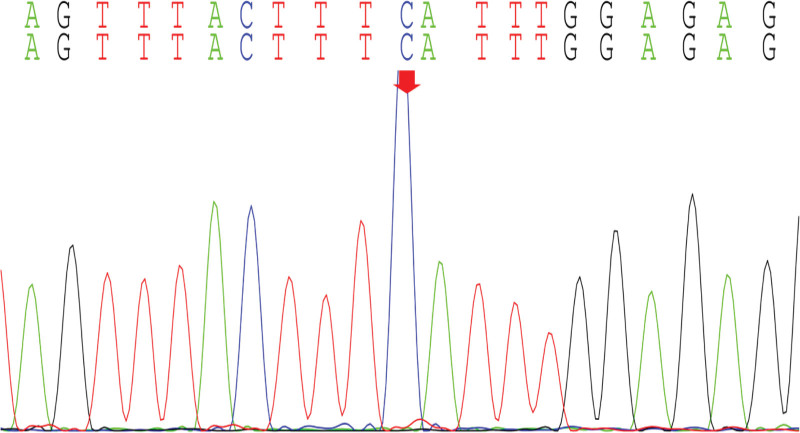
Brother of the proband.

With the informed consent of the parents of the child, the peripheral blood of the child and the parents was collected for whole exome gene sequencing, and it was found that there was a mutation in the *CWC27* gene in the child, and the mutation site was c.617(exon7)C>A (p.Ser206Ter), which was a homozygous variant, which was a pathogenic variant reported in the literature,^[3]^ and was rated pathogenic (PVS1_VeryStrong, PM2, PM3, PP3) by American College of Medical Genetics and Genomics (Figs. 8–11).

The onset age of the child is young, the course of the disease is long, and the main manifestations are unsteady walking and slow response, accompanied by typical gross motor developmental delay, special face, abnormal muscle tone, and abnormal nervous system examination. Exome sequencing found a homozygous mutation of the *CWC27* gene, combined with the clinical manifestations of the child; it was diagnosed as RPSKA.

**Figure 12.**
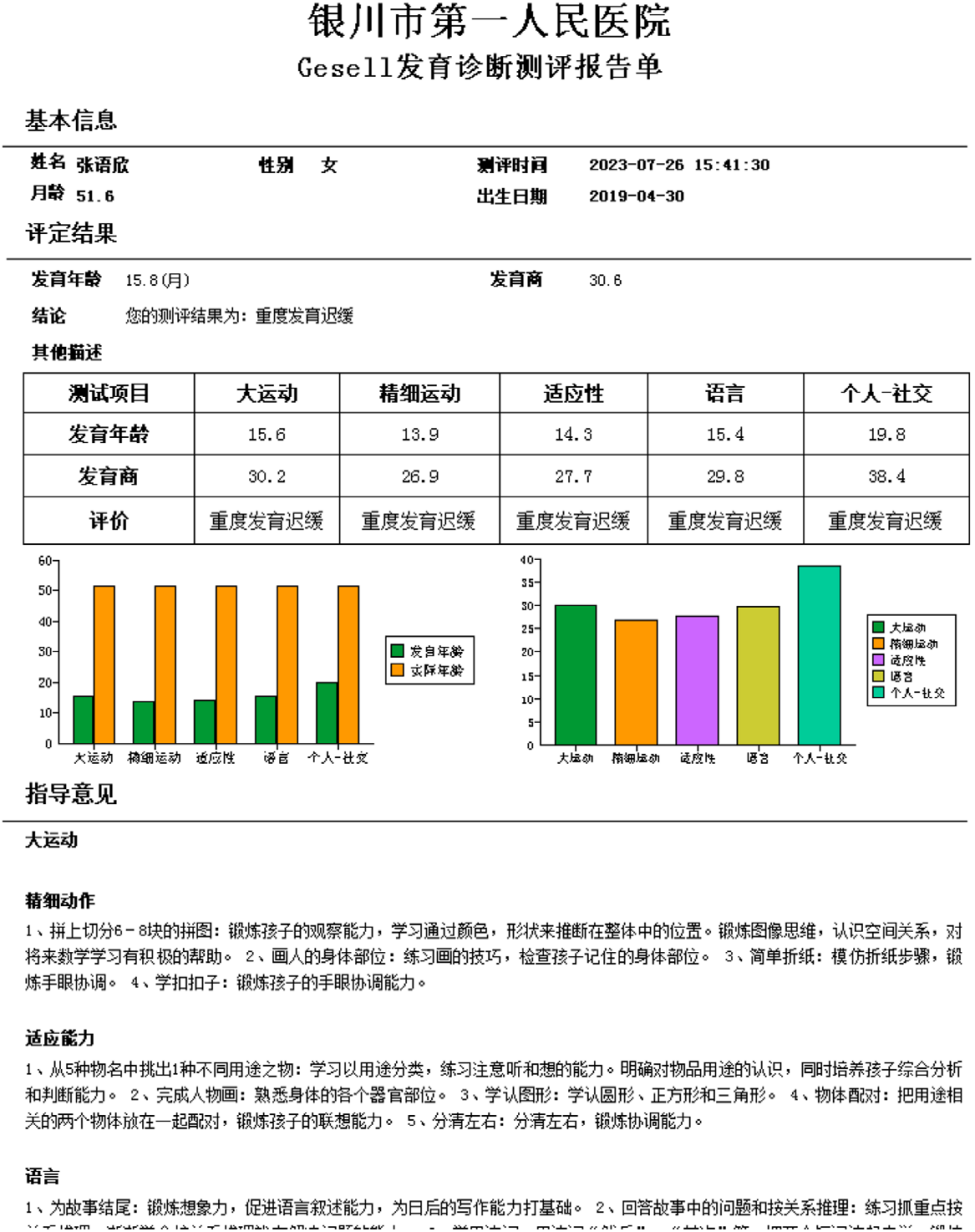
Gesell developmental assessment.

After the child was admitted to the hospital, according to the child’s symptoms and signs, we developed a detailed rehabilitation training plan, but the child’s recovery was slow, the child was 4 years and 2 months old, we conducted a comprehensive assessment of the child again, the child weighed 13.25 kg (−2SD), length 91.0 cm (−3SD), head circumference 53.5 cm, and Gesell developmental diagnosis assessment showed severe developmental delay: gross motor (30.2), fine motor (26.9), adaptability (27.7), language (29.8), and Personal—Social (36.4) (Fig. 12).

## 3. Discussion

RPSKA is a rare autosomal recessive disorder characterized by a wide variety of clinical manifestations. Early diagnosis and treatment are essential in children with developmental abnormalities, retinopathy, and skeletal disorders because of high suspicion. *CWC27* gene is a peptide-proline cis-trans isomerase (PPIase), which mediates protein–protein interactions during splice complex assembly and rearrangement and plays an important role in early tissue development and function maintenance.^[1,3,5]^

Our child was admitted to the hospital due to “unstable walking alone and slow response for more than half a year,” and the child had gross motor development delay after birth, and after a detailed physical examination at the time of admission, we found that the child had a large head circumference, high palate arch, low nose bridge, upturned nostrils, low ear position, back and other special faces, abnormal neurological examination, cranial MRI and developmental assessment suggesting developmental delay, so we highly suspected the possibility of genetic diseases in the child. After testing the whole exome gene sequencing, the gene detection showed that the *CWC27* gene was homozygous mutation, c.617(exon7)C>A (p.Ser206Ter), and the American College of Medical Genetics and Genomics guidelines determined to be a pathogenic mutation. The child’s genotype matched the clinical phenotype, and it was eventually diagnosed as RPSKA caused by homozygous mutations in the *CWC27* gene.

In addition, we would like to remind you that RPSKA is clinically similar to Bardet-Biedl syndrome, both with retinopathy and developmental delay as the main manifestations, sometimes misdiagnosed RPSKA as Bardet-Biedl syndrome; we also initially considered Bardet-Biedl syndrome, but the difference is that Bardet-Biedl syndrome is an autosomal recessive cilia caused by IFT74 gene mutations, with retinitis pigmentosa. Obesity, polydactyly, hypogonadism, and intellectual disability are characterized, and our children do not have clinical manifestations such as obesity, polydactyly, and hypogonadism.^[6–8]^

Typical symptoms of RPSKA include retinal degeneration (blepharoptosis, limited visual field, retinal vascular thinning, retinitis pigmentosa), short finger deformities (short finger/toe, shortened distal phalanx), specialized facial features (giant deformities, craniosynostosis, micro jaw deformities, low ear position), short stature, and neurodevelopmental abnormalities (intellectual development abnormalities, psychomotor retardation, feeding difficulties, language development delays, and motor development delays).^[2–4]^ Our children have special facial and neurodevelopmental abnormalities, but no typical retinopathy has been found in our children so far. This is a special feature of our children. We cannot be sure whether related lesions will appear later, and follow-up observation is still needed.

Unfortunately, there is no specific treatment for RPSKA caused by mutations in the *CWC27* gene so far, and most treatment options are based on the clinical phenotype of the child. Our children have a comprehensive developmental delay as the main performance, and we have developed a detailed rehabilitation training plan, and the children recover well after training.

## 4. Conclusion

In conclusion, we reported a case of RPSKA caused by the homozygous mutation c.617 (exon7) C>A (p.Ser206Ter) of the *CWC27* gene. Our children were mainly characterized by comprehensive developmental delay, accompanied by special facial features. However, no typical retinopathy has been detected in this child yet, and we will keep a close eye on the child for subsequent retinopathy. Our study enriched the clinical phenotype of TBL1XR1 gene mutations. The clinical phenotype of RPSKA is complex and diverse, and differential diagnosis is difficult. For children with retinopathy and skeletal development abnormalities, accompanied by special facial features, the disease is highly suspected. If necessary, further improve genetic examination to clarify the etiology, avoid misdiagnosis, and timely treatment.

## 5. Patient perspective

Although our child couldn’t be the same as other healthy peers after treatment, we have a more detailed understanding of the child’s situation with the help of doctors and have a general psychological expectation of the child’s future development. We believe that with the help of doctors, our child will have a better quality of life.

## Acknowledgments

We would like to thank the patient and her family members for their contribution to this study.

## Author contributions

**Data curation:** Jia-Yi Li.

**Formal analysis:** Jia-Yi Li.

**Project administration:** Wan-Qi Wang.

**Resources:** Wan-Qi Wang, Qian-Dui Chen.

**Software:** Qian-Dui Chen.

**Supervision:** Xiaoping Ma.

**Validation:** Xiaoping Ma.

**Visualization:** Li Liu.

**Writing – original draft:** Yang-Fan Qi.

**Writing – review & editing:** Shuang-Zhu Lin, Li Liu.
